# Drift in Diffusion Gradients

**DOI:** 10.3390/ma6083598

**Published:** 2013-08-19

**Authors:** Fabio Marchesoni

**Affiliations:** Department of Physics, University of Camerino, Camerino I-62032, Italy; E-Mail: fabio.marchesoni@pg.infn.it; Tel.: +39-320-7985898

**Keywords:** diffusion, Brownian transport, energy harvesting

## Abstract

The longstanding problem of Brownian transport in a heterogeneous quasi one-dimensional medium with space-dependent self-diffusion coefficient is addressed in the overdamped (zero mass) limit. A satisfactory mesoscopic description is obtained in the Langevin equation formalism by introducing an appropriate drift term, which depends on the system macroscopic observables, namely the diffuser concentration and current. The drift term is related to the microscopic properties of the medium. The paradoxical existence of a finite drift at zero current suggests the possibility of designing a Maxwell demon operating between two equilibrium reservoirs at the same temperature.

## 1. Introduction

Transport in heterogeneous media is characterized by microscopic mechanisms that go unnoticed under most experimental circumstances. Here I consider, for simplicity, the case of Brownian transport in a quasi one-dimensional (1D) filament directed along the *x* axis, like certain wires and narrow channels often encountered in nanotechnology and cellular biology [1]. When the characteristic length of the filament inner structure, *l*, is much shorter than the experimental resolution, Δx, of the diffusing particle coordinate, x(t), one often attempts to describe the ensuing transport process in terms of a single space-dependent mesoscopic observable, namely the transport diffusion coefficient, D(x). This coefficient is accessible to standard experimental techniques even if, in most cases, its precise relation with the particle self-diffusion coefficient in the medium, $D_{0}$, is not well established.

The local space dependence of the transport coefficient can be explained by assuming that the filament is somehow compartmentalized and particle diffusion with self-diffusion coefficient $D_{0}$ occurs in a constrained geometry [2]: the particle relaxes rapidly in each compartment, of typical size *l*, and exits it with mean first-exit time (MFET) *τ*, which depends on the geometry of the compartment itself. Under coarse graining conditions (l≪Δx), *τ* depends on the compartment coordinate *x* and so does *D*. On the other hand, the MFET to the right, τ(x+), and to the left, τ(x-), may differ, which suggests the occurrence of a local drift with velocity v(x) is proportional to -dτ(x)/dt.

The question then arises as how to formulate a stochastic description of the particle diffusion at the mesoscopic scales, Δx or larger. Due to coarse graining, viable approaches necessarily rely on phenomenological modeling, which, at best, accounts for the quantities accessible to direct observation, without assuming any particular microscopic model.

The question of the Langevin equation which best describes the Brownian motion sustained by a space-dependent noise source (multiplicative noise) dates back to the heyday of stochastic calculus [3,4,5] and is often referred to as the Itô *versus* Stratonovich dilemma [6,7].

From a mathematical viewpoint the issue was settled long ago: as proven by van Kampen [4], the dilemma is always the consequence of some sort of coarse graining scheme, where one makes use of a reduced number of stochastic variables (one in our case) to model otherwise exceedingly complex microscopic dynamics. By suitably enlarging the variable space, one avoids multiplicative noises and the dilemma is solved (see, e.g., [8]). Alternatively, one can circumvent this difficulty by introducing a coarse-grained probability density P(x,t) and modeling the process under study in the framework of the Fokker-Planck equation formalism [5]. From the starting Fokker-Planck equation (or more generally, the master equation [4]) it is possible to write down an equivalent Langevin equation according to either the Itô or Stratonovich interpretation, the difference between the two being an additional drift term, also known as Stratonovich drift [5].

From a phenomenological viewpoint, however, this dilemma keeps bothering theoreticians [9,10,11] and experimentalists [12,13,14,15] alike. The space dependence of coarse-grained transport coefficients has been experimentally confirmed for a variety of systems, whereas the direct observation of local (Stratonovich) drifts, v(x), has proved elusive. As a space-dependent D(x) hints at a multiplicative noise source, many investigators speculate that v(x) ought to follow uniquely from the knowledge of D(x), according to some “proper prescription” of stochastic calculus [4]. Unfortunately, numerical and experimental observations have yielded conflicting results, which have led to questioning the correct formulation of Fick’s law in the presence of diffusion gradients [9].

In Section 2, I show that, as anticipated by other authors [16,17,18,19], this is a moot question, as the correct answer requires a more complete description of the actual transport process on the mesoscopic scale. Indeed, under stationary conditions, local drifts are a unique function of the mesoscopic parameters one introduces to control particle transport. For the 1D filament modeled here, such parameters are the particle distribution and current, $P_{0}\left(x\right)$ and $J_{0}$ respectively, both quantities being numerically and experimentally tunable. In Section 3, I consider Brownian transport in narrow compartmentalized channels with various geometries, which result in distinct generalizations of Fick’s law. In Section 4, I discuss the implications of finite drift at zero current. In particular, I show that finite drift can be exploited to deliver cargoes and information along a filament even if it connects two equilibrium heat and (or) particle reservoirs both kept at the same temperature. In Section 5, I discuss possible applications of this effect to design a Maxwell demon capable of rectifying equilibrium thermal fluctuations.

## 2. Mesoscopic Langevin Equation

Writing the Langevin equation for Brownian transport in a heterogeneous medium with space-dependent diffusion coefficient is less a question of correctly addressing the Itô *versus* Stratonovich dilemma than of achieving a complete mesoscopic description of the process. In this section, I follow, for simplicity, the Tupper and Yang approach [19].

Tupper and Yang arguments are best illustrated for the case of a linear filament of length *L*, directed along the *x* axis, and carrying a low-density particle flow with transport diffusion coefficient D(x). Assuming coarse graining, the filament can be regarded as continuous and homogeneous in space. I further assume that both the stationary probability density in the filament, $P_{0}\left(x\right)$, and the stationary current, $J_{0}$, are known quantities (normalized to one particle). The starting point is then the Fokker-Planck equation:

(1) $$\partial_{t}P\left(x,t\right)=-\partial_{x}j\left(x,t\right)$$

with

(2) $$j\left(x,t\right)=[v\left(x\right)-\partial_{x}D\left(x\right)]P\left(x,t\right)$$

For the stationarity condition, $\lim_{t\to \infty }j\left(x,t\right)=J_{0}$, to hold, v(x) must satisfy the identity:

(3) $$v\left(x\right)=\frac{1}{P_{0}\left(x\right)}\left[J_{0}+\frac{d}{dx}\left[D\left(x\right)P_{0}\left(x\right)\right]\right]=\frac{J_{0}}{P_{0}\left(x\right)}+D\left(x\right)\frac{d}{dx}\ln\left[D\left(x\right)P_{0}\left(x\right)\right]$$

where both D(x) and $P_{0}\left(x\right)$ are definite positive.

The drift in Equation (3) consists of two terms: (i) a standard drift, $J_{0}L$, which is related to some external bias. This term will be ignored in the following by setting $J_{0}=0$; and (ii) a term of Fick’s type, which depends on D(x) and $P_{0}\left(x\right)$ but not on $J_{0}$. This is a generalized form of Stratonovich drift [4,19] and the focus of our investigation.

The drift at zero current can be explicitly calculated in two special cases:

(i) **Constant** $P_{0}\left(x\right)$: this means that the diffusing particle is uniformly distributed along the filament in spite of the space-dependence of D(x). The drift then boils down to:

(4) $$v\left(x\right)=D^{\prime }\left(x\right)$$

where the prime sign denotes a spatial derivative, and the corresponding density reads:

(5) $$j\left(x,t\right)=-D\left(x\right)\partial_{x}P\left(x,t\right)$$

(ii) **Constant** $D\left(x\right)P_{0}\left(x\right)$: this choice reflects the common expectation that under nonequilibrium conditions freely diffusing particles would condense at the colder end of the filament. This is an experimental circumstance often addressed in biological systems [9,20]. If this is the case, then:

(6) v(x)=0

and

(7) $$j\left(x,t\right)=-\partial_{x}D\left(x\right)P\left(x,t\right)$$

We have thus reproduced both options of Fick’s law generalization debated in [9]. They are not mutually exclusive, but rather model distinct diffusion conditions.

*Remark 1*—To make contact with the standard notation adopted in the classical transport literature, see e.g., [21], the current density (Equation 2) for $J_{0}=0$ can be rewritten as

(8) $$j\left(x,t\right)=\left[\mu \left(x\right)f\left(x,D_{0}\right)-D\left(x\right)\partial_{x}\right]P\left(x,t\right)$$

where

(9) $$\mu \left(x\right)=D\left(x\right)/D_{0}$$

defines the particle (quasi-equilibrium) mobility and:

(10) $$f\left(x,D_{0}\right)=D_{0}\frac{d}{dx}\left[\ln P_{0}\left(x\right)\right]$$

can be regarded as an entropic force due to coarse graining [21,22]. Whereas this formulation of j(x,t) is more common in the transport literature, application and results of the stochastic techniques introduced in Section 4 are more effectively presented in the notation of Equations (1)–(3).

*Remark 2*—To make an explicit connection with stochastic calculus, we note that v(x) in both Equations (4) and (6) can be regarded as the Stratonovich drift [5],

(11) $$v\left(x\right)=\alpha D^{\prime }\left(x\right)P\left(x,t\right)$$

associated to the phenomenological Langevin equation:

(12) $$\dot{x}=\sqrt{D(x)}\;\xi \left(t\right)$$

Here, ξ(t) is a stationary Gaussian noise with 〈ξ(t)〉=0 and 〈ξ(t)ξ(0)〉=2δ(t). The coupling between the random variable x(t) and the noise ξ(t) in the multiplicative term, $\sqrt{D(x)}\xi \left(t\right)$, depends on the stochastic calculus prescription one adopts. In the current literature [5], such prescription is defined by the parameter *α* with α∈[0,1] where α=1/2 for Stratonovich calculus, α=0 for Itô calculus, and α=1 for anti-Itô (or isothermal) calculus. The Stratonovich drift is proportional to *α*. In this notation, the current densities of Equations (5) and (7) follow immediately the multiplicative Langevin Equation (12), respectively, in the anti-Itô and Itô interpretation. Of course, this remark does not hold true for any *x* dependence of $D\left(x\right)P_{0}\left(x\right)$. In passing we also note that the stochastic processes modeled by Equation (1) [or Equation (12)] are microscopically reversible for any choice of $P_{0}\left(x\right)$ (or *α*) [18].

## 3. Drift in a Graded Channel

**Figure 1:**
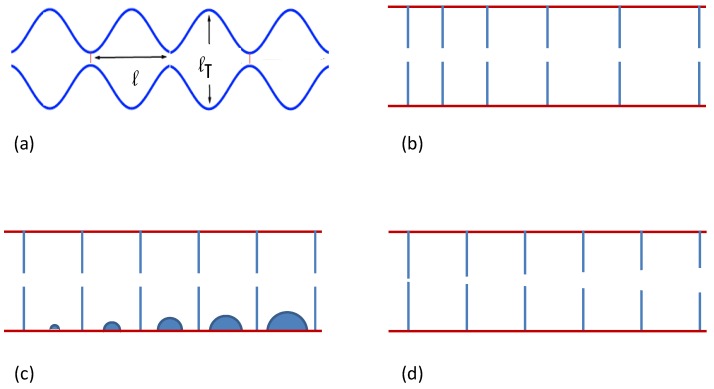
Sketch of the graded-channel geometries discussed in Section 3: (**a**) a symmetric periodic channel; (**b**) a graded compartment, length l(x); (**c**) a graded compartment, volume Ω(x); and (**d**) graded pore size.

I show now how v(x) can depend on the microscopic structure of the conducting medium. Let us consider Brownian diffusion in a narrow, two-dimensional (2D) channel mimicking our 1D filament. Let us consider here the case of smooth channel corrugation [21,22]. The channel is divided up into identical, mirror symmetric compartments with sinusoidal profile y=±w(x), where w(x)=a-bcos(2πx/l) and a>b≥0; the compartments are connected by pores with diameter Δ=2(a-b), see Figure 1a, and reflecting boundary conditions are assumed. In the absence of external drives, this 2D process can be reduced to a 1D process with probability density ρ(x,t) obeying the kinetic equation [2]:

(13) $$\begin{array}{ccc} \partial_{t}\rho \left(x,t\right) & = & \partial_{x}D_{\rho }\left(x\right)w\left(x\right)\partial_{x}\left[\rho \left(x,t\right)/w\left(x\right)\right]\\ & = & \partial_{x}\left\{-{\left[D_{\rho }\left(x\right)\ln w\left(x\right)\right]}^{\prime }+\partial_{x}D_{\rho }\left(x\right)\right\}\rho \left(x,t\right) \end{array}$$

with $D_{\rho }\left(x\right)=D_{0}/{\left[1+w^{\prime }{\left(x\right)}^{2}\right]}^{1/3}$. In deriving the Fokker-Planck Equation (13), fast intra-compartmental relaxation was assumed in the attempt to single out the inter-compartmental diffusion. The reader should not be misled by the apparent similarities of Equations (1) and (13): Equation (13) gives a full account of the microscopic diffusion process occurring inside and between adjacent compartments, whereas Equation (1) was meant to provide a mesoscopic description of the diffusion along the filament on scales much larger than the compartment length, *l*. The details of the intra- and inter-compartment diffusion are lost due to coarse graining. This is why I used a different notation for the probability density in Equation (13), namely, ρ(x,t) instead of P(x,t). To reconcile the two approaches, one should space average $D_{\rho }\left(x\right)$ and $\rho_{0}\left(x\right)=\lim_{t\to \infty }\rho \left(x,t\right)$. Both averages are uniform in *x*, which implies that for mirror symmetric periodic channels [23,24,25], v(x)=0. The corresponding mesoscopic Fokker-Planck Equation (1) describes a free Brownian motion in 1D with self-diffusion coefficient $D=\int_{0}^{l}D_{\rho }\left(x\right)dx\leq D_{0}$. The extension of the Tupper and Yang approach to the case of sharp channel corrugation [26] is straightforward. Finally, exactly the same argument applies to Büttiker’s torch model [27] (see also the discussion in [7]). Due to its spatial periodicity this model can also be coarse grained by following Section 2 Equations (2) and (3) with $P_{0}\left(x\right)$ a homogeneous function and $J_{0}\neq 0$ given by Equation (2.19) of [27].

I consider now graded, *i.e.*, non-periodic channels, with different compartment geometries.

**Figure 2:**
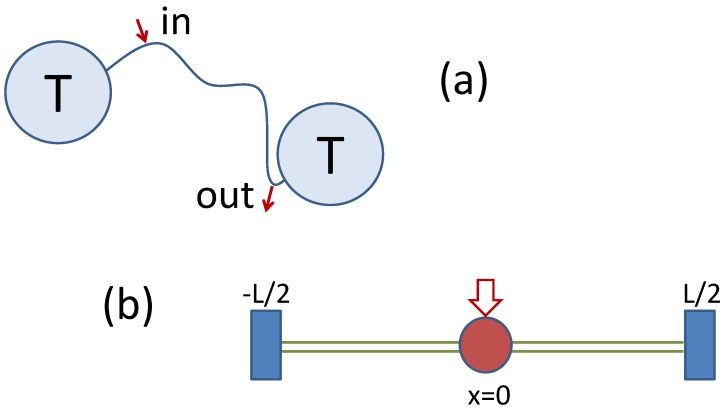
Sketch of the drift-based transport mechanism discussed in Section 4: (**a**) thin filament connecting two particle reservoirs with temperature T; and (**b**) docking (red circle) and delivery stations (blue rectangles) in a narrow channel tailored such that $v\left(x\right)=D^{\prime }\left(x\right)$.

1. Graded compartment length. I assume rectangular compartments with space-graded length l(x) and constant width $l_{⊺}$, as sketched in Figure 2b. To avoid useless mathematical complications, I further assume that all pores are identical with symmetric profile and width Δ, and neglect altogether the thickness of the dividing walls. The MFET out of a compartment centered in *x* is then [26,28,29],

(14) $$\tau \left(x\right)≃\frac{\Omega (x)}{D_{0}}\;g\left(\frac{\Delta }{l_{⊺}}\right)$$

where $\Omega \left(x\right)≃l_{⊺}l\left(x\right)$ is the compartment volume and *g* an appropriate geometric factor. The corresponding transport diffusion coefficient (for constant compartment occupancy) is then also space-dependent [29]:

(15) $$D_{\rho }\left(x\right)=\frac{l{\left(x\right)}^{2}}{4\tau (x)}≃D_{0}\;\frac{l{\left(x\right)}^{2}}{4\Omega (x)}\;g^{-1}\left(\frac{\Delta }{l_{⊺}}\right)$$

One notices immediately that Ω∝l and $D_{\rho }\propto l$. As the 2D particle density is constant everywhere, the effective probability density $P_{0}\left(x\right)∼\Omega \left(x\right)/l\left(x\right)$ is *x* independent and so is the averaged diffusion coefficient, $D\left(x\right)\propto D_{\rho }\left(x\right)/\Omega \left(x\right)$. As a consequence, the Stratonovich drift of Equation (3) vanishes, *i.e.*, v(x)=0. This conclusion applies for any l(x) as long as the filament thickness is small, $l_{⊺}\ll \Delta x$, and homogeneous in *x*.

2. Graded compartment volume. I consider now the case of rectangular compartments of the same length, *l*, width, $l_{⊺}$, and with identical symmetric pores, as described in item 1, see Figure 1c. The main difference consists in the small obstructions of varying volume ω(x) pressed against the channel walls. Their exact position is irrelevant as long as $\omega \ll l_{⊺}l$. Here, again, the microscopic 2D particle density is constant, so that $P_{0}\left(x\right)\propto \Omega \left(x\right)$ with $\Omega \left(x\right)=l_{⊺}l-\omega \left(x\right)$. From Equation (15), $D_{\rho }\propto \Omega^{-1}$ and, after averaging, $D\propto \Omega^{-2}$. As a consequence, the Stratonovich drift of Equation (3) turns out to be finite and space dependent, namely:

(16) $$v\left(x\right)\propto -\Omega^{\prime }\left(x\right)/\Omega {\left(x\right)}^{3}\propto \omega^{\prime }\left(x\right)$$

the second relation holding for $\omega \left(x\right)\ll l_{⊺}l$.

3. *Graded compartment pores.* I now assume that all compartments are rectangular with volume $\Omega =l_{⊺}l$; only the width of the pores, Δ(x), varies with *x*, see Figure 1d. In this case $P_{0}\left(x\right)$ is uniform in space, contrary to D(x), which is proportional to $D_{\rho }\left(x\right)$ and, in view of Equation (15), a function of Δ(x). On applying the relevant expression for the drift in Equation (4), I finally obtain:

(17) $$v\left(x\right)=D^{\prime }\left(x\right)=-D\left(x\right)\frac{d}{dx}\ln\left[g\left(\frac{\Delta (x)}{l_{⊺}}\right)\right]$$

A generalization of the rate theory arguments used above also provides a qualitative interpretation of the analytical expressions for D(x) and v(x) derived in Section 2. On assuming coarse graining, I have been regarding a particle of coordinate *x* as confined to a microscopic compartment of arbitrarily short length *l* and centered at *x*. This defines two distinct MFET’s, one to the right (left pore closed) τ(x+), and one to the left (right pore closed) τ(x-). Correspondingly, the MFET for the particle to diffuse out of the compartment in either direction can be expressed as [29] τ(x)=τ(x-)τ(x+)/[τ(x-)+τ(x+)]. For compartments of *constant* length much smaller the spatial resolution, l≪Δx, the exit times τ(x±) are equal in the leading order, independent of the compartment asymmetry; hence, the approximate identities τ(x)≃τ(x+)≃2τ(x) and, as anticipated in Equation (15), $D\left(x\right)=l^{2}/4\tau \left(x\right)$. The space-dependent D(x) is thus a *local* quantity, insensitive to the compartment asymmetry.

Compartment asymmetry, instead, is responsible for the Stratonovich drift. In the absence of external bias, $J_{0}=0$, a drift emerges due to the difference between the average compartment traversal velocities to the right and to the left, namely:

(18) $$v\left(x\right)=\frac{l}{P_{0}\left(x-\right)+P_{0}\left(x+\right)}\;\left[\frac{P_{0}\left(x-\right)}{\tau (x+)}-\frac{P_{0}\left(x+\right)}{\tau (x-)}\right]$$

Here, I weighted the velocities $l/\tau (x_{\pm })$ with respect to the probability densities, $P_{0}\left(x_{\mp }\right)$, of the drifting particle. Once more, coarse graining approximations, 2τ(x)≃τ(x)≃τ(x+) and $P_{0}\left(x\right)≃P_{0}\left(x_{-}\right)≃P_{0}\left(x_{+}\right)$, can be invoked to rewrite Equation (18) as:

(19) $$v\left(x\right)=\frac{l^{2}}{4P_{0}\left(x\right)}\;\frac{d}{dx}\frac{P_{0}\left(x\right)}{\tau (x)}=\frac{1}{P_{0}\left(x\right)}\;\frac{d}{dx}\left[D\left(x\right)P_{0}\left(x\right)\right]$$

which coincides with the prescription of Equation (3) for $J_{0}=0$. Although I have obtained the present estimates for D(x) and v(x) under the assumption that *l* is space-homogeneous, the ensuing mesoscopic description is independent of the details of the underlying microscopic model. The corresponding coarse grained dynamics is encoded by the Langevin equation [5]:

(20) $$\dot{x}=-v\left(x\right)+\sqrt{D(x)}\;\xi \left(t\right)$$

where ξ(t) is defined in Equation (12) and, due to the local (or non-advancing [5]) nature of D(x), the multiplicative noise has to be interpreted *à la* Itô. As a consequence, the Langevin Equation (20) encodes the same dynamics as the Fokker-Planck Equations (1) and (2).

## 4. Applications to Transport

I discuss now in some details the properties of a mesoscopic Fokker-Planck equation with finite drift of the type introduced in Equation (4) and exemplified in item 3 of the previous section, namely $v\left(x\right)=D^{\prime }\left(x\right)$. Such a Fokker-Planck equation can be rewritten as:

(21) $$\partial_{t}P\left(x,t\right)=\partial_{x}D\left(x\right)\partial_{x}P\left(x,t\right)$$

see Equation (8), and describes diffusion conditions frequently encountered in the classical transport literature (see, e.g., [14,15]). As an example inspired by cellular biology, I consider a filament of length *L* connecting two heat reservoirs in equilibrium at the same temperature, see Figure 2a. The diffusion process described by Equation (21) is then restricted, say to the interval (-L/2,L/2); it admits uniform stationary probability density, $P_{0}\left(x\right)$ and zero current, $J_{0}=0$. If the inner structure of the filament is compartmentalized as in Figure 1d, the question arises of how the finite Stratonovich drift can be experimentally demonstrated.

To this purpose I propose the following thought experiment: a docking station is placed at the filament midpoint, x=0, where (non-obtrusive) cargoes can be randomly loaded on a diffusing particle which happens to pass by. Alternatively, one can imagine storing an information bit on the particle, say, by orienting its spin. Two delivery stations at the filament endpoints x=±L/2 are equipped to download the cargo (or read out the information) as the particle *first* reaches them. I regard these two operations respectively as particle tagging and un-tagging. The question asked here is: what are the probabilities, $\Pi_{\pm }$, for the cargo to be delivered at ±L/2? And, related to this question, what are the corresponding delivery times, $T_{\pm }\left(0\right)$? The main conclusion of this exercise is that for a positive drift $\Pi_{+}>\Pi_{-}$ and $T_{+}\left(0\right)<T_{-}\left(0\right)$. Note that this results hold in the presence of *zero current*.

To answer the first question I make use of the notion of splitting probabilities detailed in Chapter 5 of [5]. The probabilities $\Pi_{\pm }\left(x\right)$, with $\Pi_{-}+\Pi_{+}=1$, that a Brownian particle obeying the Fokker-Planck Equation (21), injected at the point of coordinate *x* in the interval (-L/2,L/2), reaches first ±L/2 are, respectively,

(22) $$\begin{array}{ccc} \Pi_{-} & = & I/(1+I)\\ \Pi_{+} & = & 1/(1+I) \end{array}$$

with

(23) $$I\left(x\right)=\frac{\Pi_{-}\left(x\right)}{\Pi_{+}\left(x\right)}=\frac{\int_{x}^{L/2}dy/\psi \left(y\right)}{\int_{-L/2}^{x}dy/\psi \left(y\right)}$$

(See Equations (5.2.189) and (5.2.190) of [5], where, to make contact with the present problem, one must set ψ(x)=D(x).) In a numerical simulation $\Pi_{\pm }\left(0\right)$ can be determined by injecting $N_{t}$ particles at x=0 and counting the number $N_{\pm }$ of those exiting the filament first to the right (left). Of course, $N_{-}+N_{+}=N_{t}$ and the ratio $N_{-}/N_{+}$ coincides with the quantity *I* introduced in Equation (23).

To answer the second question I make use of the following MFET’s known from the literature:

(i) MFET to the right (reflecting left end, absorbing right end):

(24) $${\tilde{T}}_{+}\left(x\right)=\int_{x}^{L/2}\frac{dy}{\psi (y)}\int_{-L/2}^{y}\frac{\psi (z)}{D(z)}dz$$

(ii) MFET to the left (reflecting right end, absorbing left end):

(25) $${\tilde{T}}_{-}\left(x\right)=\int_{-L/2}^{x}\frac{dy}{\psi (y)}\int_{y}^{L/2}\frac{\psi (z)}{D(z)}dz$$

(iii) MFET through either side (two absorbing ends):

(26) $$T\left(x\right)=\Pi_{-}\left(x\right){\tilde{T}}_{-}\left(x\right)+\Pi_{+}\left(x\right){\tilde{T}}_{+}\left(x\right)-\Pi_{-}\left(x\right)\Pi_{+}\left(x\right)\int_{-L/2}^{L/2}\frac{dy}{\psi (y)}\times \int_{-L/2}^{L/2}\frac{\psi (z)}{D(z)}dz$$

(See Equations (5.2.160), (5.2.161) and (9.1.24) of [5].)

The MFET’s I want to calculate obey the identity:

(27) $$T\left(x\right)=\Pi_{-}\left(x\right)T_{-}\left(x\right)+\Pi_{+}\left(x\right)T_{+}\left(x\right)$$

which, combined with Equation (26), yields:

(28) $$T_{\pm }\left(x\right)={\tilde{T}}_{\pm }\left(x\right)-\frac{\Pi_{\mp }\left(x\right)}{2}\int_{-L/2}^{L/2}\frac{dy}{\psi (y)}\times \int_{-L/2}^{L/2}\frac{\psi (z)}{D(z)}dz$$

and, finally,

(29) $$T_{+}\left(x\right)=\frac{1}{2}\int_{x}^{L/2}\frac{dy}{\psi (y)}\left[\int_{-L/2}^{y}\frac{\psi (z)}{D(z)}dz-\int_{y}^{L/2}\frac{\psi (z)}{D(z)}dz\right]$$

(30) $$T_{-}\left(x\right)=\frac{1}{2}\int_{-L/2}^{x}\frac{dy}{\psi (y)}\left[\int_{y}^{L/2}\frac{\psi (z)}{D(z)}dz-\int_{-L/2}^{y}\frac{\psi (z)}{D(z)}dz\right]$$

These last two formulas can be readily specialized for ψ(x)=D(x), to obtain:

(31) $$T_{+}\left(x\right)=\int_{x}^{L/2}\frac{y}{D(y)}dy,\;\;\;\;\;\;\;\;\;T_{-}\left(x\right)=-\int_{-L/2}^{x}\frac{y}{D(y)}dy$$

that is, $T_{+}\left(x\right)-T_{-}\left(x\right)=\int_{-L/2}^{L/2}\;ydy/D\left(y\right)$

As a check of this result, I consider the case when the space-dependence of the transport diffusion coefficient can be linearized as $D\left(x\right)=D_{0}+\epsilon x/L$, with ϵ=D(L/2)-D(-L/2) quantifying the filament asymmetry. Straightforward calculations lead to:

(32) $$I\left(0\right)=\frac{\Pi_{-}\left(0\right)}{\Pi_{+}\left(0\right)}=-\frac{\ln\left(1+\frac{\epsilon }{2D_{0}}\right)}{\ln\left(1-\frac{\epsilon }{2D_{0}}\right)}≃1-\frac{\epsilon }{2D_{0}}$$

and

(33) $$T_{\pm }\left(0\right)=\frac{L^{2}}{2\epsilon }\left[1\mp \frac{2D_{0}}{\epsilon }\ln\left(1\pm \frac{\epsilon }{2D_{0}}\right)\right]≃\frac{L^{2}}{8D_{0}}\left(1\mp \frac{\epsilon }{3D_{0}}\right)$$

the approximate equalities holding for small asymmetries, $\epsilon \ll D_{0}$.

In conclusion, the particles injected at the loading station placed along the filament reach the delivery station on the right with higher probability and faster. This effect takes advantage of the local drift imposed by the channel asymmetry upon particles—the carriers—otherwise diffusing with zero current under equilibrium conditions. The role of D(x) space-dependence is apparent in Equations (32) and (33). Accordingly, one can easily prove that for v(x)=0—see item 1 of Section 2—$T_{+}\left(0\right)=T_{-}\left(0\right)$, as expected.

## 5. Conclusions

Numerical simulations, not reported here, confirm that the diffusion of overdamped Brownian particles confined to the narrow asymmetric channel of Section 3, item 3, does produce a uniform equilibrium distribution with no net current, while the individual particles exhibit a drift in the direction of the gradient, $D^{\prime }\left(x\right)$, of the transport diffusion coefficient, *i.e.*, to the right. This situation of “drift without flux” is well explained by the authors of [17]. Note that the rectification effect discussed in Section 4 should not be mistaken for just another ratchet effect. To overcome the restrictions imposed by the second law of thermodynamics, two basic ingredients (Pierre Curie’s conjecture) are generally required to operate a rectifier as a ratchet [30]: spatial asymmetry of the substrate and time correlation of the fluctuations, random or deterministic, applied to the diffusing particles. The second requirement clearly indicates that a ratchet works under nonequibrium conditions.

Contrary to the ratchet prescriptions, in the thought experiment of Section 3 a net cargo (information) flow from the loading to the right hand side delivery station, with failure rate $\Pi_{-}/\Pi_{+}$, was established by taking advantage of Brownian diffusion in *thermal equilibrium*. According to Curie’s conjecture, surely some time-symmetry breaking mechanism must have been at work, lest one violated the second law of thermodynamics [30]. As a matter of fact, tagging a particle at x=0 and un-tagging it at x=±L/2 has a two-fold consequence: (i) Only particles initially contained in a restricted portion of the filament are utilized. These particles then diffuse in time according to a *non-stationary process homogeneous* with the equilibrium process described by the Fokker-Planck Equation (21) [5]. This takes care of Curie’ conjecture. (ii) The average drift, 〈v(x)〉, is a transient observable. Out of the entire equilibrium ensemble of stochastic trajectories, only those originated at x=0 and ending at x=±L/2 are selected. Do these trajectories provide free cargo or information shipping along the filament? To answer this question, one must consider that a “Maxwell demon” capable of tagging and un-tagging a diffusing particle, wastes a finite amount of energy (or free-energy), even in the ideal case when it does so without perturbing the particle dynamics. The efficiency of the demon operation can be easily estimated in the formalism of [31]. Let $\gamma =kT/D_{0}$ denote the (large) viscous constant of a particle diffusing in a fluid at temperature *T*. For the process of Section 4 the particle drifts along the filament with average velocity $\langle v\rangle =\left(L/N_{t}\right)\left(N_{+}/T_{+}-N_{-}/T_{-}\right)$. The work done to transport a cargo a distance *L* is thus $W_{cargo}=\gamma \langle v\rangle L$. The minimum energy, $W_{load}$ required to tag and un-tag a diffuser is kTln2 for each operation, as established by the so-called Landauer principle [32]. The efficiency of this transient rectification process is therefore:

$$\eta =W_{cargo}/W_{load}\propto \epsilon /D_{0}$$

where ϵ=D(L/2)-D(-L/2) and $D\left(x\right)\propto D_{0}$, *η* is a small but finite quantity independent of the heat bath parameters *γ* and *T*. The Maxwell demon of Section 4 harnesses equilibrium fluctuations during a transient to transport cargoes (information) over finite distances: it pays for loading (and unloading) but not for shipping.
